# Prediction of cardiovascular markers and diseases using retinal fundus images and deep learning: a systematic scoping review

**DOI:** 10.1093/ehjdh/ztae068

**Published:** 2024-09-10

**Authors:** Livie Yumeng Li, Anders Aasted Isaksen, Benjamin Lebiecka-Johansen, Kristian Funck, Vajira Thambawita, Stine Byberg, Tue Helms Andersen, Ole Norgaard, Adam Hulman

**Affiliations:** Department of Public Health, Aarhus University, Bartholins Allé 2, 8000 Aarhus C, Denmark; Steno Diabetes Center Aarhus, Aarhus University Hospital, Palle Juul-Jensens Boulevard 11, 8200 Aarhus N, Denmark; Steno Diabetes Center Aarhus, Aarhus University Hospital, Palle Juul-Jensens Boulevard 11, 8200 Aarhus N, Denmark; Steno Diabetes Center Aarhus, Aarhus University Hospital, Palle Juul-Jensens Boulevard 11, 8200 Aarhus N, Denmark; Steno Diabetes Center Aarhus, Aarhus University Hospital, Palle Juul-Jensens Boulevard 11, 8200 Aarhus N, Denmark; Department of Holistic Systems, SimulaMet, Stensberggata 27, 0170 Oslo, Norway; Clinical Epidemiological Research, Copenhagen University Hospital — Steno Diabetes Center Copenhagen, Borgmester Ib Juuls Vej 83, 2730 Herlev, Denmark; Department of Education, Danish Diabetes Knowledge Center, Copenhagen University Hospital — Steno Diabetes Center Copenhagen, Borgmester Ib Juuls Vej 83, 2730 Herlev, Denmark; Department of Education, Danish Diabetes Knowledge Center, Copenhagen University Hospital — Steno Diabetes Center Copenhagen, Borgmester Ib Juuls Vej 83, 2730 Herlev, Denmark; Department of Public Health, Aarhus University, Bartholins Allé 2, 8000 Aarhus C, Denmark; Steno Diabetes Center Aarhus, Aarhus University Hospital, Palle Juul-Jensens Boulevard 11, 8200 Aarhus N, Denmark

**Keywords:** Cardiovascular disease, Cardiovascular risk, Retinal fundus image, Deep learning, Clinical prediction, Systematic scoping review

## Abstract

Rapid development in deep learning for image analysis inspired studies to focus on predicting cardiovascular risk using retinal fundus images. This scoping review aimed to identify and describe studies using retinal fundus images and deep learning to predict cardiovascular risk markers and diseases. We searched MEDLINE and Embase on 17 November 2023. Abstracts and relevant full-text articles were independently screened by two reviewers. We included studies that used deep learning for the analysis of retinal fundus images to predict cardiovascular risk markers or cardiovascular diseases (CVDs) and excluded studies only using predefined characteristics of retinal fundus images. Study characteristics were presented using descriptive statistics. We included 24 articles published between 2018 and 2023. Among these, 23 (96%) were cross-sectional studies and eight (33%) were follow-up studies with clinical CVD outcomes. Seven studies included a combination of both designs. Most studies (96%) used convolutional neural networks to process images. We found nine (38%) studies that incorporated clinical risk factors in the prediction and four (17%) that compared the results to commonly used clinical risk scores in a prospective setting. Three of these reported improved discriminative performance. External validation of models was rare (21%). There is increasing interest in using retinal fundus images in cardiovascular risk assessment with some studies demonstrating some improvements in prediction. However, more prospective studies, comparisons of results to clinical risk scores, and models augmented with traditional risk factors can strengthen further research in the field.

## Graphical Abstract

**Graphical Abstract:**
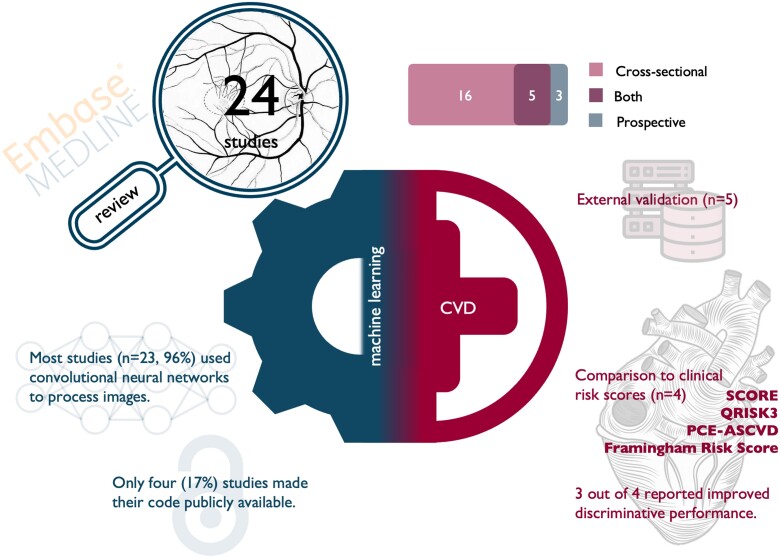

## Introduction

Cardiovascular diseases (CVDs) are the leading causes of mortality globally.^1^ Clinical risk prediction models can help to identify individuals at high risk and target preventive efforts including lifestyle and pharmacological interventions.^2^ There are some well-established and validated CVD prediction models based on socio-demographic factors and traditional clinical variables in the general population and in the selected subpopulations, like the Framingham score,^3^ SCORE2,^4^ and QRISK models.^5^ A recent comprehensive systematic review called for evaluating the predictive performance of existing tools in different populations, tailoring existing models to specific populations, and identifying new data sources to be included in models, instead of testing small alterations of the established clinical risk models.^6^

Developments in machine learning for image analysis in the last decade have made it feasible to include images as a data type in risk prediction models, potentially in combination with traditional risk factors (multimodal prediction models).^7,8^ Retinal fundus imaging is a relatively simple, low-cost, and the only non-invasive method used for assessing the state of blood vessels in the body.^9^ People with type 1 and type 2 diabetes are regularly invited to diabetic retinopathy screening with retinal fundus images, which makes it a potentially relevant predictor and allows us to follow the progression of microvascular disease.^10^ It’s likely that the underlying disease causes damage to all blood vessels, resulting in both micro- and macrovascular complications.^11^ The state of the blood vessels in the retina could reflect that of blood vessels elsewhere.^12,13^ There is a well-established association between diabetic retinopathy and both micro- and macrovascular complications, including CVD.^14,15^ However, it remains an open question whether these associations can be translated into clinically relevant predictors. Instead of using disease status as the predictor, the actual images might carry more relevant information for CVD prediction.

In a preliminary search, we identified a review on the topic of our scoping review.^16^ However, there are some limitations of this work. Firstly, the search procedure was lacking details. Secondly, the authors only considered open-access articles in the review. Thirdly, they had a focus on diabetic retinopathy grading and image segmentation unrelated to CVD. Since the review was published, we have identified several relevant articles on the topic.^17,18^ Finally, we aim to evaluate the studies from a more clinical perspective (e.g. performance comparison with or added value to existing risk scores).

We chose to conduct a systematic scoping review instead of a classical systematic review because scoping reviews serve to scope a body of literature, examine research practices, and clarify concepts, which fit well with our goals.^19^ We aimed to describe studies, with an emphasis on clinical perspectives, that used retinal fundus images and deep learning for the prediction of cardiovascular markers and diseases.

## Methods

The scoping review was conducted in accordance with the Joanna Briggs Institute methodology for scoping reviews and reported according to the Preferred Reporting Items for Systematic Reviews and Meta-analyses extension for scoping review guidelines.^20^ The scoping review protocol was published on Figshare on 19 December 2023.^21^

### Eligibility criteria

#### Participants

We considered studies analysing data from human participants regardless of their health status (e.g. population-based studies or cohorts with a specific condition).

#### Context

This scoping review focuses on studies in the clinical research context, regardless of the geographical location, ethnicity, and gender composition of the study populations. Methodological articles comparing different methods for CVD risk prediction based on retinal fundus images were considered if any of the methods used deep learning.

#### Types of sources

We included peer-reviewed articles (e.g. original articles, brief reports, and peer-reviewed full-length articles in conference proceedings). Included studies needed to be human clinical studies (including methodological studies with examples using clinically relevant outcomes and measures). Furthermore, we only included studies that used deep learning to predict cardiovascular markers, or presence of CVD, or CVD incidence based on retinal fundus images. Studies could also use a deep learning–derived score from retinal fundus images (not pre-defined retinal features), to predict cardiovascular markers or diseases.

We excluded review articles, editorials, conference abstracts, and preprints. Studies were excluded if they only included optical coherence tomography or other eye images for prediction, if they extracted pre-defined features of the vessel network (e.g. tortuosity and fractal dimension) and then associated them with CVD markers as deep learning methods are less relevant for these studies, or if they predicted factors that were not based on measurements of the cardiovascular system, but merely risk factors of CVD (e.g. age, sex, cholesterol, and HbA1c). We also excluded association studies, even if they investigated the association between a deep learning–derived score and cardiovascular markers or diseases, since such studies do not report any predictive performance metrics.

Articles in languages not understood by the review team (English, Danish, Swedish, Norwegian, Hungarian, German, Polish, and Chinese) that were considered eligible by title and abstract were not included in the synthesis but would have been included in an appendix for others to analyse.

### Search strategy

We considered MEDLINE and Embase as databases for the search. Most of the data science literature focuses on diabetic retinopathy grading, most likely due to the availability of open-access data sets from this domain. As this task is not directly relevant from a cardiovascular perspective and it is unlikely that such data sets have detailed phenotyping of cardiovascular health or follow-up for hard endpoints, we did not consider technical databases.

Information specialist T.H.A. conducted the search on 17 November 2023. The search comprised three key concepts: retina, CVDs, and artificial intelligence (AI)/machine learning. Each concept was searched using Medical Subject Headings and free-text words, and no limits were applied. The search string was developed in MEDLINE and subsequently translated to Embase. The search string was tested against eight key articles within the field and reviewed by another information specialist (O.N.). The full search string in both databases is available on Figshare.^22^

After the selection process, we used the software tool citationchaser^23^ to retrieve all references within and all articles citing the included articles and previous reviews within the same topic. Retrieved articles were screened to find all relevant articles.

### Study selection

Following the search, all identified citations were collated and uploaded into EPPI-Reviewer 6 and duplicates were removed.^24^

In the screening phase, two independent reviewers screened titles and abstracts to assess eligibility. To clarify screening criteria and increase consistency, we had a pilot screening workshop examining the inclusion and exclusion of 25 abstracts. In the official screening, the reviewers met to assess alignment in the process after screening 10% of the abstracts. After all titles and abstracts had been screened, full-text versions of relevant articles were retrieved and assessed in detail against the eligibility criteria by two or more independent reviewers. Reasons for the exclusion of articles at full-text screening were recorded and reported in the Results section. Any disagreements between the reviewers at any stage of the selection process were resolved through discussion. If there was no consensus after this, the senior author (A.H.) made the final decision. The study selection process is presented using a flow diagram.

### Data extraction

Research questions (*Table 1*) were pre-specified and published in the scoping review protocol.^21^ Data were extracted from the included articles by the first author and verified by the senior author. Data was collected using a data extraction instrument developed based on the research questions. The extracted data included specific details about the study methods and characteristics relevant to the review questions listed in the protocol (e.g. first author and year of publication; study population and design; CVD outcomes; deep learning model used; predictive performance; and comparison with clinical risk scores if included). The identified studies described in the data extraction table are published on Figshare.^22^

**Table 1:** *A priori* defined research questions

1	To what extent is deep learning used for predicting cardiovascular risk using retinal fundus images and what characterizes the studies?
2	Which cardiovascular markers and diseases are predicted in the studies?
3	What are the study populations (e.g. general population, type 2 diabetes)?
4	How diverse are study populations (age, sex, ethnicity, geographic location)?
5	How do prediction models perform? Which clinical prediction performance metrics are used?
6	Are deep learning models compared with classical clinical risk prediction models?
7	Are the models validated internally or externally?
8	Are the models tested or implemented in a clinical setting?
9	What kind of deep learning architectures are used (e.g. convolutional neural networks)?
10	In the case of incident cardiovascular disease as the outcome (follow-up studies), how is the time-to-event nature of data handled?
11	Are there studies combining other data modalities (e.g. tabular/structured clinical data) with retinal fundus images?
12	Are there any publicly available data sets (open/restricted by payment/private)?
13	Are the models and the code publicly available (e.g. on Hugging Face or GitHub)?

### Data analysis

Study characteristics are aggregated using descriptive statistics (frequencies and percentages). A narrative summary accompanies the tabulated and charted results and describes how the results relate to the review’s objective and questions.

## Results

**Figure 1:**
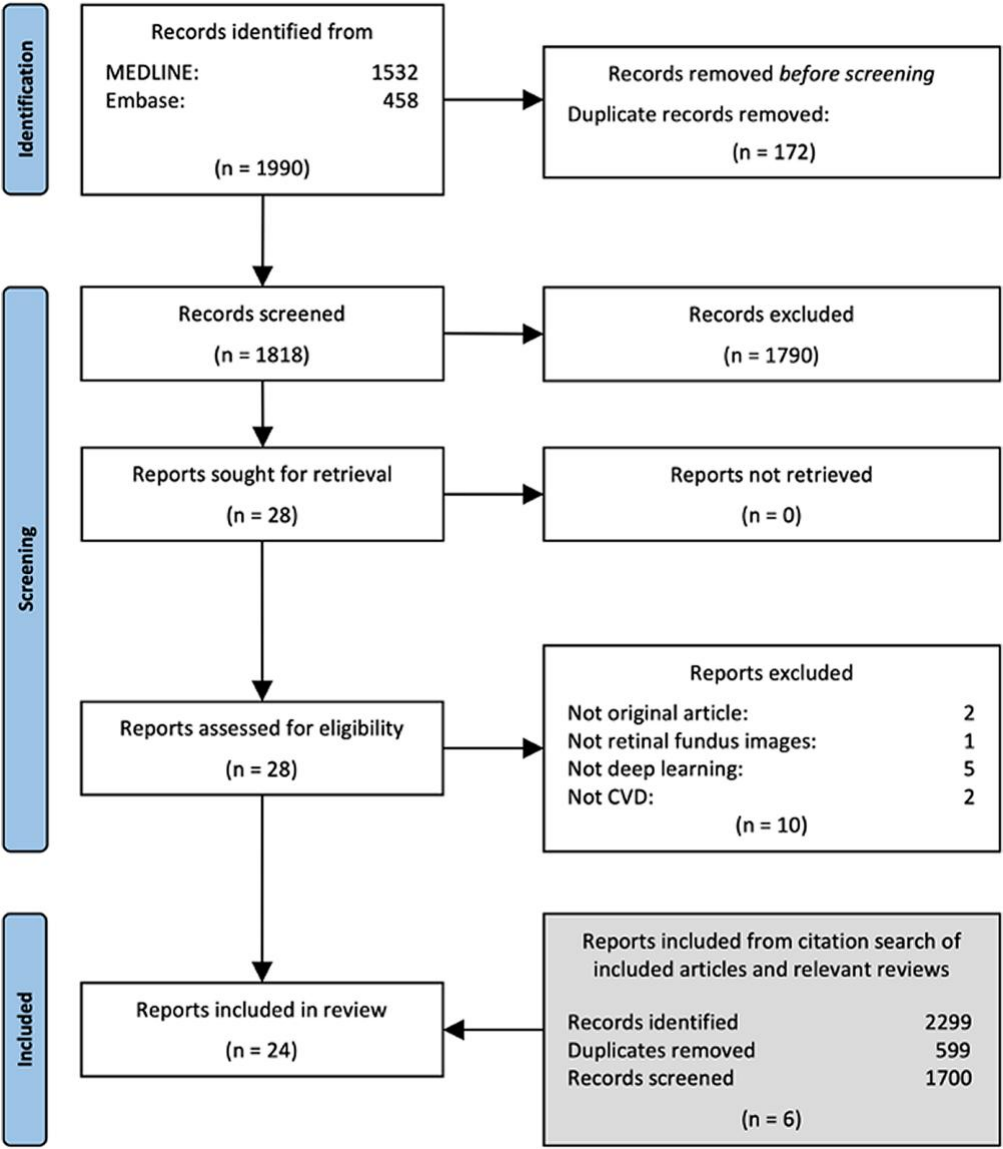
PRISMA flow chart.

The search resulted in 1990 records, of which 172 were duplicates (*Figure 1*). After screening the titles and abstracts of the remaining 1818 records, 1790 were excluded as irrelevant. Of the 28 records included for full-text screening, 10 were excluded because they did not use deep learning (*n* = 5), were not original articles (*n* = 2), did not focus on CVD (*n* = 2), or did not use fundus retinal images (*n* = 1). Another six records were identified in citing articles and references of the included studies and previous reviews on similar topics.^16,25^ In total, 24 studies were included in this scoping review, all of which were published after 2018 (*Table 2*).

**Table 2:** Study design and outcome of included studies

Article	Follow-up time	Country	Recruitment period	Number of participants	Number of events/cases	Main outcome
Cross-sectional studies						
Al-Absi *et al*.^26^		QA	2012–not specified	483	233	Diagnosis of cardiovascular disease
Barriada *et al.*^27^		ES	2014–2018	76	33	Coronary artery calcium score (>400/<400)
Chang *et al*.^28^		KR	2005–2016	6579	N/A	Carotid intima-media thickness
Cho *et al*.^29^		KR	2010–2017	2696	Not reported	Coronary artery calcium score (>100/<100)
Coronado *et al*.^30^		UK	2006–2010	2472	412	Stroke
Dai *et al*.^31^		CN	2017–2018	1419	735	Hypertension
Diaz-Pinto *et al*.^32^		UK	2006–2010	5663	N/A	Left ventricular characteristics
Ding *et al*.^33^		CN	2018–2021	25 222	10 225	Coronary heart disease
Gerrits *et al*.^34^		QA	2012–not specified	3000	N/A	Systolic and diastolic blood pressure
Jeena *et al*.^35^		IN	Not specified	130^a^	50^a^	Stroke
Lee *et al*.^17^		KR	2010–2016	2026	50%^b^	Coronary heart disease, cerebrovascular disease
Li *et al*.^36^		CN	Not specified	250	100	Stroke
Lim *et al*.^37^		SG	Not specified	11 150^a,c^	4528^a,c^	Stroke
Mellor *et al*.^38^		UK	2005–2017	226 855^c^	N/A	Systolic and diastolic blood pressure
Mueller *et al*.^39^		DE	Not specified	77	57	Peripheral arterial disease
Nagasato *et al*.^40^		JP	2018–2019	85	N/A	Brachial-ankle pulse wave velocity
Poplin, *et al*.^41^		UK	2006–2010	48 101	N/A	Systolic and diastolic blood Pressure
Rim *et al*.^42^		KR	Not specified	34 395	N/A	Systolic and diastolic blood pressure
Rim *et al*.^43^		KR	Not specified	11 243^c^	N/A	Coronary artery calcium score (0/0–100/>100)
Son *et al*.^44^		KR	2003–2016	20 130	19 176	Coronary artery calcium score (>100/<100)
Zhang *et al*.^45^		CN	2017	625	253	Hypertension
Follow-up studies						
Chang *et al.*^28^	7.6 y (median)	KR	2005–2016	32 227	78	Cardiovascular disease mortality
Diaz-Pinto *et al*.^32^	Not specified	UK	2006–2010	71 515	992	Myocardial infarction
Lee *et al*.^46^	5.0 y (up to)	KR	2013–2018	1106	33	Cardiovascular disease mortality, major heart failure, stroke, myocardial infarction, coronary artery calcium score (0/0–100/>100)^d^
Mellor *et al*.^38^	4–9 y (median)	UK	2005–2017	226 855^c^	40 802^c^	Peripheral arterial disease, coronary heart disease, stroke, myocardial infarction
Poplin *et al*.^41^	Not specified	UK	2006–2010	11 835	105	Major adverse cardiovascular events
Rim *et al*.^43^	4.1 y (median)	KR	Not specified	527	33	Cardiovascular disease mortality
	10 y (median)	SG	Not specified	8551	310	Cardiovascular disease mortality
	10 y (median)	UK	Not specified	47 679	337	Cardiovascular disease mortality
Tseng *et al*.^47^	11 y (median)	UK	2009–2010	48 260	2766	Cardiovascular disease mortality, stroke, coronary heart disease, coronary artery calcium score (0/0–100/>100)^d^
Zhou *et al*.^48,^e^^	Not specified	UK	2000–2022	8110	4055	Major heart failure
	Not specified	UK	2000–2022	2526	1263	Stroke
	Not specified	UK	2000–2022	2806	1403	Myocardial infarction

^a^Number of patients was not reported, but the number of retinal images was reported in the study.

^b^Number of patients was not reported, but the percentage of images from patients with outcome diseases was reported in the study.

^c^Multiple study populations were included in the study; the total number of patients was reported here.

^d^Cross-sectional outcome.

^e^Extra study populations were included for the development of the foundation model in the study.

Among the identified studies, 13 out of 24 (54%) predicted markers of subclinical CVD as the outcome, 18 predicted clinical CVD, and 7 studies included both types of outcomes. Ten out of 18 studies were cross-sectional using retinal fundus images to predict prevalent CVD, and 8 were cohort studies including clinical CVD (see Supplementary material online, *Table S1*).

Markers of subclinical CVD included systolic and diastolic blood pressure, left ventricular characteristics, brachial-ankle pulse wave velocity, coronary artery calcium (CAC) score, and carotid intima-media thickness (see Supplementary material online, *Table S1*). Clinical CVD included hypertension, peripheral arterial disease, coronary heart disease, cerebrovascular disease, stroke, myocardial infarction, heart failure, and CVD mortality. Apart from the desired outcomes listed in the studies, one study had an outcome described as CVD without further details, and one study had the outcome described as major adverse cardiovascular events (MACEs) without further details included.

Most studies were conducted in the general population or without the mention of specific patient groups (22 out of 24). Two studies had more specific study populations, one included people with atrial fibrillation,^36^ and another included people with type 1 diabetes or type 2 diabetes.^38^ We used the first authors’ first affiliation as a proxy for the geographical location of the studies. More than half of the identified studies were conducted in Asia (13 out of 24, 54%): five from South Korea, four from China, and four from Singapore. We did not identify any studies from Africa, South America, or Australia.

Most studies (*n* = 23, 96%) used convolutional neural networks to process images. One study used the vision transformer deep learning architecture, which is a variant of the same method that is behind large language models. Instead of sequences made of words, a vision transformer processes sequences made of patches of segmented images.^48^ No studies used a deep learning framework specifically developed for time-to-event data. Several performance metrics were used in the studies. The most often used predictive performance metric was discrimination, characterized by the area under the receiver operating characteristic curve (AUROC), also referred to as C-statistic or concordance index in time-to-event settings (*n* = 18 out 24, 75%). For continuous outcomes, the most often used metrics were accuracy (*n* = 6, 25%), mean absolute error, and the coefficient of determination also known as *r*-squared (*n* = 3).

We found nine studies that combined clinical risk factors (in tabular data form) with images as predictors in their studies. Five out of nine included clinical risk factors in the deep learning models, whereas the other four applied a two-step approach extracting new variables using deep learning and then including them in Cox regression or Poisson regression models. Five studies compared the performance with established clinical risk scores such as Framingham Risk Score,^49^ Systematic Coronary Risk Assessment (SCORE),^50^ QRISK3,^51^ and the Pooled Cohort Equation for Atherosclerotic CVD (PCE-ASCVD) (*Table 3*).^52^ One study presented a comparison with a customized clinical risk prediction model developed as part of the same study.^38^ Four studies reported incremental improvement in the predictive performance after adding retinal fundus images or scores derived from them to the clinical risk score or model of their choice.

**Table 3:** Characteristics of studies that compared retina-based prediction models with established clinical cardiovascular risk scores

First author, year	Validation, model	Outcome, main results
Follow-up studies		
Poplin, 2018^41^	Internal split CNN^a^ (Inception-v3)	5-year major adverse cardiovascular events Systematic Coronary Risk Assessment (SCORE): AUROC = 0.72 (0.67, 0.76) SCORE + retinal images: AUROC = 0.72 (0.67, 0.76) No improvement with adding algorithm
Chang, 2020^28^	Internal split CNN (Xception model) + Cox regression	Cardiovascular disease mortality Framingham Risk Score (FRS): AUROC = 0.78 (0.73, 0.82) FRS + retinal images: AUROC = 0.81 (0.76, 0.85) Improvement with adding images: ΔAUROC = 0.0266 (0.0043, 0.0489) *P* = 0.02
Rim, 2021^43^	Internal split and external CNN (EfficientNet)	Cardiovascular disease events in SCORE outcome Pooled Cohort Equation (PCE): AUROC = 0.595 (0.572, 0.618) PCE + RetiCAC: AUROC = 0.626 (0.595, 0.657) Improvement with adding images: ΔAUROC = 0.031 (0.010, 0.051) *P* = 0.0036
Tseng, 2023^47^	Not applicable (validation study) CNN + Cox regression	10-year cardiovascular disease (non-statin group) QRISK3: AUROC = 0.682 (0.672, 0.692) QRISK3 + RetiCAC: AUROC = 0.696 (0.686, 0.706) Improvement with adding images: ΔAUROC = 0.014 (0.010, 0.017) *P* < 0.001 (stage 1 hypertension cohort) QRISK3: AUROC = 0.639 (0.620, 0.658) QRISK3 + RetiCAC: AUROC = 0.652 (0.633, 0.671) Improvement with adding images: ΔAUROC = 0.013 (0.007, 0.019) *P* < 0.001 (middle-aged cohort) QRISK3: AUROC = 0.650 (0.638, 0.662) QRISK3 + RetiCAC: AUROC = 0.674 (0.661, 0.686) Improvement with adding images: ΔAUROC = 0.023 (0.018, 0.029) *P* < 0.001
Cross-sectional studies		
Lee, 2023^17^	Internal split and external CNN (DenseNet-169)	Prevalent cardiovascular disease (case-control) Pooled Cohort Equation: AUROC = 0.677 (0.658, 0.696) Clinical risk factors + retinal images: AUROC = 0.872 (0.857, 0.886)

^a^CNN, convolutional neural network.

External validation of models was rare (*n* = 5 out of 24, 21%),^17,32,42,43,48^ while internal validation was a common practice. Eight studies used cross-validation,^26,27,30,31,35,39,40,44^ and 12 split and set part of their study sample aside for validation.^17,28,29,33,34,37,38,41–43,45,48^ No study used a fully open-access data set. Eight studies used data from the UK Biobank,^53^ which is available to anyone for a data access fee.^17,30,32,41–43,47,48^ Only four studies made their code publicly available on GitHub.^32,39,42,48^ None of the studies reported impact studies to evaluate the implementation of the developed algorithms and scores.

## Discussion

We identified and described studies exploring the integration of retinal fundus images in the prediction of cardiovascular markers and diseases. The majority of these studies were cross-sectional, lacking examination of predictive utility for hard clinical endpoints in prospective settings. Few studies compared their models with established cardiovascular risk scores, evaluating the potential of the multimodal approaches. In most of these cases, including retina images led to some improvements in discriminative performance. However, other clinically relevant metrics, like calibration, were often overlooked.

Clinical risk prediction models are traditionally developed using regression-based statistical methods that can only handle categorical and numerical variables, i.e. tabular data. Recent advancements in deep learning allowed the analysis and integration of images with promising results in various medical domains, including clinical risk assessment of cardiovascular health.^54,55^ A multimodal approach offers the opportunity to extract novel insights from new, and often routinely collected data types, expanding the utilization of available clinical data.

We found a series of studies on prediction of CAC from one research group, where they developed and validated a retinal fundus image-based score referred to as Reti-CVD or RetiCAC, and its utility was compared with the existing CVD risk scores.^43,46,47^ The score was proposed as a non-invasive and cost-efficient alternative to the computer tomography–derived CAC score. Multiple validation studies investigated the incremental predictive value of RetiCAC on top of the established clinical risk prediction models. Rim *et al*.^43^ added the score to PCE-ASCVD to predict SCORE CVD events, and Tseng *et al*.^47^ added the score to QRISK3 to predict 10-year CVD, respectively. They both reported some improvements in predictive performance after adding retinal image-derived scores to the models. In another study, Reti-CVD was used to identify people at intermediate and high risk of CVD according to established clinical risk scores (PCE-ASCVD, QRISK3, and Framingham Risk Score) and reported that it could effectively identify these groups.^18^ Lee *et al*.^46^ conducted a regulated pivotal trial (single-centre conformity design and confirmatory retrospective analysis) to validate the efficacy of Reti-CVD for stratification of CVD risk. The trial concluded superior performance in risk stratification compared with some subclinical CVD markers (carotid intima media thickness and pulse wave velocity) and non-inferiority to CAC score-based risk stratification. The commercialized product based on the RetiCAC/Reti-CVD score obtained CE approval as a class IIa medical device in the European Union and several countries in Asia, which is the only product we identified in our search. In South Korea, patients also have a formal process to get reimbursement for Reti-CVD.^56^ However, our search has not identified any impact studies conducted to evaluate the actual clinical impact of using the score either on clinical care or patient outcomes.

In addition to the studies focusing on CAC prediction, three other studies compared their predictive performance with established clinical risk scores. Poplin *et al*.^41^ used a deep learning model to predict the 5-year MACE from retinal images. They compared the predictive performance of the original SCORE model with its image-augmented version. They did not find evidence for improved predictive performance by including retinal images. Chang *et al*.^28^ had a similar approach to the outcome of CVD mortality using the Framingham Risk Score. This study reported minor improvements by integrating images into the model. In a case-control study, Lee *et al*.^17^ predicted the prevalence of CVD using a multimodal deep learning model integrating retinal images and traditional risk factors and compared the results with the predictive ability of PCE-ASCVD. They found major performance improvement by using the deep learning model.

When examining performance evaluations, we found a strong focus on model discrimination, which describes how well a model ranks predicted probabilities between individuals with and without an event. However, in a clinical setting, CVD risk scores are often used with a specific threshold (e.g. 10%) to make decisions on interventions, e.g. treatment intensification. Therefore, good discrimination alone is not sufficient, but calibration is highly relevant as well, which was overlooked by most of the studies. A miscalibrated model can lead to resource misallocation. Individuals incorrectly predicted with a high risk of CVD may be administered unnecessary tests or treatments, while those who could benefit from interventions may be overlooked.

Apart from the use of clinically relevant performance metrics for model evaluation, fair comparisons between existing and new models are also of high importance. Some of the identified studies reported that retinal images can be used to predict demographic factors (e.g. age and sex) and traditional clinical risk factors (e.g. blood pressure and body mass index).^41^ Many of these factors are associated with CVD risk; therefore, good performance of a prediction model based only on images does not necessarily mean that retinal images have additional value for CVD risk prediction, which is the underlying motivation of most identified studies. Models might achieve good performance by indirectly predicting risk factors that are easier to collect than retinal images.^41^ This question can be partly addressed by stratifying participants by clinical risk factors possible to predict based on retinal images (e.g. age and sex).^34^ Another solution is to conduct incremental comparisons integrating images with traditional clinical risk factors; however, one should be careful with interpreting the results if important, unobserved risk factors are not included in the models.

Although studies demonstrated the potential of deep learning to contribute to improvements in cardiovascular risk assessment, methodological challenges, like insufficient validation and poor reproducibility due to the lack of openness about data and code, must be addressed by future studies. Moreover, diverse study populations, both for development and validation, would improve the equality and fairness of potential clinical applications.^57,58^ We could not identify any impact studies in our scoping review, which is a crucial missing link to measure the actual clinical utility of retinal image-based prediction models in CVD risk assessment. This is in line with cardiovascular research in general, where impact studies are rare.^59,60^

Although most deep learning models were originally not designed to analyse time-to-event data, recent developments enabled them to account for this special data structure.^61,62^ However, none of the identified studies applied such a time-to-event framework. The most common approach to circumvent this limitation was a two-step approach combining deep learning with classical survival analysis methods.^38,43,46,47^ In step one, the deep learning model takes one or a pair of retinal fundus images as input and outputs an intermediate score or abstract feature representation. In step two, the score is used, often in combination with clinical risk factors, as input for a Cox or Poisson regression model, that then outputs predicted probabilities. Since these statistical models are consistent with what is used in classical survival analyses, the comparison and validation are analogous to established risk scores and risk prediction models. Some studies ignored the time-to-event nature of the data, which can lead to underestimation of CVD risk especially in high-risk groups.^63^

### Strengths and limitations

As a systematic scoping review, the strength of our study is the comprehensive and systematic exploration of the scope. The search strategy was thoroughly developed and documented contributing to transparency and reproducibility. Our review was written with a broad target audience addressing study characteristics relevant from both a clinical and a data science perspective. One limitation of our study is that we searched only medical databases and might have overlooked studies from the technical sciences and engineering communities. To address this, we screened all references and citing articles of the included studies and reviews similar to ours. As a result, we included six additional articles, five of which were not indexed in the medical databases we searched.

### Perspectives

In this systematic scoping review, we identified a gap between the proposed methods in clinical research and the latest developments in the field of deep learning. Retina fundus imaging as a non-invasive, low-cost method generates images that contain information on cardiovascular health that can be extracted using deep learning. Combining the images with clinical risk factors could improve early detection of high-risk individuals and facilitate intervention to prevent CVD morbidity and mortality. A fully deep learning–driven multimodal time-to-event prediction model could bring new insight to CVD progression and risk analysis by learning from a series of images in longitudinal settings. Our findings support that improvements are needed in the recently proposed five critical quality criteria for AI-based prediction model development and validation studies.^64^ Research in this highly interdisciplinary field must strive to report adequate details from both the clinical and data science aspects to document the clear intended use in clinical practice. Further collaboration and better communication between the two fields are crucial to raising the quality of studies and ensuring the alignment between deep learning–driven approaches and clinical needs and standards. More validation studies with a focus on diversity regarding geographical location and ethnicity should be conducted in the future. At the same time, efforts should be spent to promote open-access data sets and algorithms to align with the FAIR principles.^65^ With the new AI Act approved in the European Parliament and similar proposals introduced globally, it is expected that research utilizing AI technology will be forced to adhere to stricter regulations regarding transparency, accountability, and ethical considerations throughout the developmental and implementation lifecycle.

## Conclusion

Our scoping review presented the landscape of how deep learning methods integrate retinal fundus images in cardiovascular risk prediction. Retinal fundus imaging offers a simple and non-invasive method to assess vascular health. Advances in the field of AI offer the potential to integrate this knowledge into risk prediction and to extend existing risk assessment models based on traditional clinical risk factors. Some evidence shows improvements in predictive performance when adding images to clinical risk factors, and one commercialized product is approved as software as a medical device in several countries and regions. To further strengthen research in the field, there is a need to promote the use of other clinically relevant performance metrics than discrimination, such as calibration, increase efforts on external validation of findings in data sets from diverse settings, and initiate prospective studies to evaluate the impact of new multimodal risk assessment tools.

## Supplementary Material

ztae068_Supplementary_Data

## Data Availability

The study protocol and the completed data extraction forms were published elsewhere (see Methods section).

## References

[1] GBD 2017 Causes of Death Collaborators . Global, regional, and national age-sex-specific mortality for 282 causes of death in 195 countries and territories, 1980–2017: a systematic analysis for the Global Burden of Disease study 2017. Lancet 2018;392:1736–1788. 30496103 10.1016/S0140-6736(18)32203-7 PMC6227606

[2] Bruninx A, Scheenstra B, Dekker A, Maessen J, van ‘t Hof A, Kietselaer B, et al Using clinical prediction models to personalise lifestyle interventions for cardiovascular disease prevention: a systematic literature review. Prev Med Rep 2022; 25:101672. 35127352 10.1016/j.pmedr.2021.101672 PMC8800044

[3] D’Agostino RB, Vasan RS, Pencina MJ, Wolf PA, Cobain M, Massaro JM, et al General cardiovascular risk profile for use in primary care. Circulation 2008;117:743–753. 18212285 10.1161/CIRCULATIONAHA.107.699579

[4] SCORE2 working group and ESC Cardiovascular risk collaboration . SCORE2 risk prediction algorithms: new models to estimate 10-year risk of cardiovascular disease in Europe. Eur Heart J 2021;42:2439–2454. 34120177 10.1093/eurheartj/ehab309 PMC8248998

[5] Hippisley-Cox J, Coupland C, Vinogradova Y, Robson J, May M, Brindle P. Derivation and validation of QRISK, a new cardiovascular disease risk score for the United Kingdom: prospective open cohort study. BMJ 2007;335:136. 17615182 10.1136/bmj.39261.471806.55 PMC1925200

[6] Damen JAAG, Hooft L, Schuit E, Debray TPA, Collins GS, Tzoulaki I, et al Prediction models for cardiovascular disease risk in the general population: systematic review. BMJ 2016;353:i2416. 27184143 10.1136/bmj.i2416 PMC4868251

[7] Topol EJ . As artificial intelligence goes multimodal, medical applications multiply. Science 2023;381:eadk6139. 10.1126/science.adk6139 37708283

[8] Acosta JN, Falcone GJ, Rajpurkar P, Topol EJ. Multimodal biomedical AI. Nat Med 2022;28:1773–1784. 36109635 10.1038/s41591-022-01981-2

[9] Suzuki Y . Direct measurement of retinal vessel diameter: comparison with microdensitometric methods based on fundus photographs. Surv Ophthalmol 1995;39:S57–S65. 7660313 10.1016/s0039-6257(05)80074-8

[10] American Diabetes Association Professional Practice Committee . 12. retinopathy, neuropathy, and foot care: *standards of Medical Care in Diabetes—*2022. Diabetes Care 2022;45:S185–S194. 34964887 10.2337/dc22-S012

[11] Fowler MJ . Microvascular and macrovascular complications of diabetes. Clin Diabetes 2008;26:77–82.

[12] Hanssen H, Streese L, Vilser W. Retinal vessel diameters and function in cardiovascular risk and disease. Prog Retin Eye Res 2022;91:101095. 35760749 10.1016/j.preteyeres.2022.101095

[13] Seidelmann SB, Claggett B, Bravo PE, Gupta A, Farhad H, Klein BE, et al Retinal vessel calibers in predicting long-term cardiovascular outcomes: the atherosclerosis risk in communities study. Circulation 2016;134:1328–1338. 27682886 10.1161/CIRCULATIONAHA.116.023425 PMC5219936

[14] Pearce I, Simó R, Lövestam-Adrian M, Wong DT, Evans M. Association between diabetic eye disease and other complications of diabetes: implications for care. A systematic review. Diabetes Obes Metab 2019;21:467–478. 30280465 10.1111/dom.13550 PMC6667892

[15] Xie J, Ikram MK, Cotch MF, Klein B, Varma R, Shaw JE, et al Association of diabetic macular edema and proliferative diabetic retinopathy with cardiovascular disease. JAMA Ophthalmia 2017;135:586–593. 10.1001/jamaophthalmol.2017.0988 PMC5593137 28472362

[16] Barriada RG, Masip D. An overview of deep-learning-based methods for cardiovascular risk assessment with retinal images. Diagnostics 2023;13:68. 10.3390/diagnostics13010068 PMC9818382 36611360

[17] Lee YC, Cha J, Shim I, Park WY, Kang SW, Lim DH, et al Multimodal deep learning of fundus abnormalities and traditional risk factors for cardiovascular risk prediction. Npj Digit Med 2023;6:14. 36732671 10.1038/s41746-023-00748-4 PMC9894867

[18] Yi JK, Rim TH, Park S, Kim SS, Kim HC, Lee CJ, et al Cardiovascular disease risk assessment using a deep-learning-based retinal biomarker: a comparison with existing risk scores. Eur Heart J—Digit Health 2023;4:236–244. 37265875 10.1093/ehjdh/ztad023 PMC10232236

[19] Munn Z, Peters MDJ, Stern C, Tufanaru C, McArthur A, Aromataris E. Systematic review or scoping review? Guidance for authors when choosing between a systematic or scoping review approach. BMC Med Res Methodol 2018;18:143. 30453902 10.1186/s12874-018-0611-x PMC6245623

[20] Tricco AC, Lillie E, Zarin W, O’Brien KK, Colquhoun H, Levac D, et al PRISMA extension for scoping reviews (PRISMA-ScR): checklist and explanation. Ann Intern Med 2018;169:467–473. 30178033 10.7326/M18-0850

[21] Li LY, Isaksen AA, Lebiecka-Johansen B, Funck KL, Thambawita V, Byberg S, et al Prediction of cardiovascular markers and diseases using retinal fundus images and deep learning: a systematic scoping review protocol. Figshare 2023. 10.6084/m9.figshare.24793203.v1 (19 December 2023, 24 June 2024). PMC11570365 39563905

[22] Li LY, Isaksen AA, Lebiecka-Johansen B, Funck KL, Thambawita V, Byberg S, et al Supplementary Materials for Prediction of Cardiovascular Markers and Diseases Using Retinal Fundus Images and Deep Learning: A Systematic Scoping Review. Figshare 2024. 10.6084/m9.figshare.25610088.v1 (17 April 2024, 24 June 2024). PMC11570365 39563905

[23] Haddaway NR, Grainger MJ, Gray CT. Citationchaser: a tool for transparent and efficient forward and backward citation chasing in systematic searching. Res Synth Methods 2022;13:533–545. 35472127 10.1002/jrsm.1563

[24] Thomas J, Graziosi S, Brunton J, Ghouze Z, O’Driscoll P, Bond M, et al EPPI-Reviewer: advanced software for systematic reviews, maps and evidence synthesis. London: EPPI-Centre, UCL Social Research Institute, University College London; 2022.

[25] Hu W, Yii FSL, Chen R, Zhang X, Shang X, Kiburg K, et al A systematic review and meta-analysis of applying deep learning in the prediction of the risk of cardiovascular diseases from retinal images. Transl Vis Sci Technol 2023;12:14. 10.1167/tvst.12.7.14 PMC10353749 37440249

[26] Al-Absi HRH, Islam MT, Refaee MA, Chowdhury MEH, Alam T. Cardiovascular disease diagnosis from DXA scan and retinal images using deep learning. Sensors 2022;22:4310. 35746092 10.3390/s22124310 PMC9228833

[27] Barriada RG, Simó-Servat O, Planas A, Hernández C, Simó R, Masip D. Deep learning of retinal imaging: a useful tool for coronary artery calcium score prediction in diabetic patients. Appl Sci 2022;12:1401.

[28] Chang J, Ko A, Park SM, Choi S, Kim K, Kim SM, et al Association of cardiovascular mortality and deep learning-funduscopic atherosclerosis score derived from retinal fundus images. Am J Ophthalmol 2020;217:121–130. 32222370 10.1016/j.ajo.2020.03.027

[29] Cho S, Song SJ, Lee J, Song J, Kim MS, Lee M, et al Predicting coronary artery calcium score from retinal fundus photographs using convolutional neural networks. In: *Artificial intelligence and soft computing* 2020, Zakopane, Poland. p. 599–612. Springer.

[30] Coronado I, Abdelkhaleq R, Yan J, Marioni SS, Jagolino-Cole A, Channa R, et al Towards stroke biomarkers on fundus retinal imaging: a comparison between vasculature embeddings and general purpose convolutional neural networks. In: *2021 43rd annual international conference of the IEEE engineering in medicine & biology society (EMBC)* 2021. p. 3873–3876. IEEE. 10.1109/EMBC46164.2021.9629856 PMC8981508 34892078

[31] Dai G, He W, Xu L, Pazo EE, Lin T, Liu S, et al Exploring the effect of hypertension on retinal microvasculature using deep learning on East Asian population. PLoS One 2020;15:e0230111. 32134976 10.1371/journal.pone.0230111 PMC7058325

[32] Diaz-Pinto A, Ravikumar N, Attar R, Suinesiaputra A, Zhao Y, Levelt E, et al Predicting myocardial infarction through retinal scans and minimal personal information. Nat Mach Intell 2022;4:55–61.

[33] Ding YD, Zhang Y, He LQ, Fu M, Zhao X, Huang LK, et al A deep-learning model for the assessment of coronary heart disease and related risk factors via the evaluation of retinal fundus photographs. Zhonghua Xin Xue Guan Bing Za Zhi 2022;50:1201–1206. 36517441 10.3760/cma.j.cn112148-20221010-00783

[34] Gerrits N, Elen B, Craenendonck TV, Triantafyllidou D, Petropoulos IN, Malik RA, et al Age and sex affect deep learning prediction of cardiometabolic risk factors from retinal images. Sci Rep 2020;10:9432. 32523046 10.1038/s41598-020-65794-4 PMC7287116

[35] Jeena RS, Shiny G, Sukesh Kumar A, Mahadevan K. A comparative analysis of stroke diagnosis from retinal images using hand-crafted features and CNN. Journal of Intelligent and Fuzzy Systems 2021;41:5327–5335.

[36] Li H, Gao M, Song H, Wu X, Li G, Cui Y, et al Predicting ischemic stroke risk from atrial fibrillation based on multi-spectral fundus images using deep learning. Front Cardiovasc Med 2023;10:1185890. 37600060 10.3389/fcvm.2023.1185890 PMC10434281

[37] Lim G, Lim ZW, Xu D, Ting DSW, Wong TY, Lee ML, et al Feature isolation for hypothesis testing in retinal imaging: an ischemic stroke prediction case study. Proc AAAI Conf Artif Intell 2019;33:9510–9515.

[38] Mellor J, Jiang W, Fleming A, McGurnaghan SJ, Blackbourn L, Styles C, et al Can deep learning on retinal images augment known risk factors for cardiovascular disease prediction in diabetes? A prospective cohort study from the national screening programme in Scotland. Int J Med Inf 2023;175:105072. 10.1016/j.ijmedinf.2023.105072 37167840

[39] Mueller S, Wintergerst MWM, Falahat P, Holz FG, Schaefer C, Schahab N, et al Multiple instance learning detects peripheral arterial disease from high-resolution color fundus photography. Sci Rep 2022;12:1389. 35082343 10.1038/s41598-022-05169-z PMC8792038

[40] Nagasato D, Tabuchi H, Masumoto H, Kusuyama T, Kawai Y, Ishitobi N, et al Prediction of age and brachial-ankle pulse-wave velocity using ultra-wide-field pseudo-color images by deep learning. Sci Rep 2020;10:19369. 33168888 10.1038/s41598-020-76513-4 PMC7652944

[41] Poplin R, Varadarajan AV, Blumer K, Liu Y, McConnell MV, Corrado GS, et al Prediction of cardiovascular risk factors from retinal fundus photographs via deep learning. Nat Biomed Eng 2018;2:158–164. 31015713 10.1038/s41551-018-0195-0

[42] Rim TH, Lee G, Kim Y, Tham YC, Lee CJ, Baik SJ, et al Prediction of systemic biomarkers from retinal photographs: development and validation of deep-learning algorithms. Lancet Digit Health 2020;2:e526–e536. 33328047 10.1016/S2589-7500(20)30216-8

[43] Rim TH, Lee CJ, Tham YC, Cheung N, Yu M, Lee G, et al Deep-learning-based cardiovascular risk stratification using coronary artery calcium scores predicted from retinal photographs. Lancet Digit Health 2021;3:e306–e316. 33890578 10.1016/S2589-7500(21)00043-1

[44] Son J, Shin JY, Chun EJ, Jung KH, Park KH, Park SJ. Predicting high coronary artery calcium score from retinal Fundus images with deep learning algorithms. Transl Vis Sci Technol 2020;9:28. 10.1167/tvst.9.2.28 PMC7410115 33184590

[45] Zhang L, Yuan M, An Z, Zhao X, Wu H, Li H, et al Prediction of hypertension, hyperglycemia and dyslipidemia from retinal fundus photographs via deep learning: a cross-sectional study of chronic diseases in central China. PLoS One 2020;15:e0233166. 32407418 10.1371/journal.pone.0233166 PMC7224473

[46] Lee CJ, Rim TH, Kang HG, Yi JK, Lee G, Yu M, et al Pivotal trial of a deep-learning-based retinal biomarker (Reti-CVD) in the prediction of cardiovascular disease: data from CMERC-HI. J Am Med Inform Assoc 2023;31:130–138. 37847669 10.1093/jamia/ocad199 PMC10746299

[47] Tseng RMWW, Rim TH, Shantsila E, Yi JK, Park S, Kim SS, et al Validation of a deep-learning-based retinal biomarker (Reti-CVD) in the prediction of cardiovascular disease: data from UK Biobank. BMC Med 2023;21:28. 36691041 10.1186/s12916-022-02684-8 PMC9872417

[48] Zhou Y, Chia MA, Wagner SK, Ayhan MS, Williamson DJ, Struyven RR, et al A foundation model for generalizable disease detection from retinal images. Nature 2023;622:156–163. 37704728 10.1038/s41586-023-06555-x PMC10550819

[49] Ankle Brachial Index Collaboration . Ankle brachial index combined with Framingham risk score to predict cardiovascular events and mortality: a meta-analysis. JAMA 2008;300:197. 18612117 10.1001/jama.300.2.197 PMC2932628

[50] Conroy R . Estimation of ten-year risk of fatal cardiovascular disease in Europe: the SCORE project. Eur Heart J 2003;24:987–1003. 12788299 10.1016/s0195-668x(03)00114-3

[51] Hippisley-Cox J, Coupland C, Brindle P. Development and validation of QRISK3 risk prediction algorithms to estimate future risk of cardiovascular disease: prospective cohort study. BMJ 2017;357:j2099. 28536104 10.1136/bmj.j2099 PMC5441081

[52] Goff DC, Lloyd-Jones DM, Bennett G, Coady S, D’Agostino RB, Gibbons R, et al 2013 ACC/AHA guideline on the assessment of cardiovascular risk. J Am Coll Cardiol 2014;63:2935–2959. 24239921 10.1016/j.jacc.2013.11.005 PMC4700825

[53] Chua SYL, Thomas D, Allen N, Lotery A, Desai P, Patel P, et al Cohort profile: design and methods in the eye and vision consortium of UK Biobank. BMJ Open 2019;9:e025077. 10.1136/bmjopen-2018-025077 PMC6398663 30796124

[54] Muse ED, Topol EJ. Transforming the cardiometabolic disease landscape: multimodal AI-powered approaches in prevention and management. Cell Metab 2024;36:670–683. 38428435 10.1016/j.cmet.2024.02.002 PMC10990799

[55] Singh M, Kumar A, Khanna NN, Laird JR, Nicolaides A, Faa G, et al Artificial intelligence for cardiovascular disease risk assessment in personalised framework: a scoping review. EClinicalMedicine 2024;73:102660. 38846068 10.1016/j.eclinm.2024.102660 PMC11154124

[56] Mediwhale . Reti-CVD. https://mediwhale.com/reti-cvd/ (24 Jun 2024).

[57] Varma T, Jones CP, Oladele C, Miller J. Diversity in clinical research: public health and social justice imperatives. J Med Ethics 2023;49:200–203. 35428737 10.1136/medethics-2021-108068

[58] Dwork C, Hardt M, Pitassi T, Reingold O, Zemel R. Fairness through awareness. In: Proceedings of the 3rd Innovations in Theoretical Computer Science Conference at Cambridge Massachusetts, USA in 2012. Association for Computing Machinery. doi:10.48550/arXiv.1104.3913.

[59] Usher-Smith JA, Silarova B, Schuit E, Moons G, Griffin K, J S. Impact of provision of cardiovascular disease risk estimates to healthcare professionals and patients: a systematic review. BMJ Open 2015;5:e008717. 10.1136/bmjopen-2015-008717 PMC4636662 26503388

[60] Ban JW, Chan MS, Muthee TB, Paez A, Stevens R, Perera R. Design, methods, and reporting of impact studies of cardiovascular clinical prediction rules are suboptimal: a systematic review. J Clin Epidemiol 2021;133:111–120. 33515655 10.1016/j.jclinepi.2021.01.016

[61] Lee C, Yoon J, van der Schaar M. Dynamic-DeepHit: a deep learning approach for dynamic survival analysis with competing risks based on longitudinal data. IEEE Trans Biomed Eng 2020;67:122–133. 30951460 10.1109/TBME.2019.2909027

[62] Vale-Silva LA, Rohr K. Long-term cancer survival prediction using multimodal deep learning. Sci Rep 2021;11:13505. 34188098 10.1038/s41598-021-92799-4 PMC8242026

[63] Li Y, Sperrin M, Ashcroft DM, Van Staa TP. Consistency of variety of machine learning and statistical models in predicting clinical risks of individual patients: longitudinal cohort study using cardiovascular disease as exemplar. BMJ 2020;371:m3919. 33148619 10.1136/bmj.m3919 PMC7610202

[64] Van Royen FS, Asselbergs FW, Alfonso F, Vardas P, Van Smeden M. Five critical quality criteria for artificial intelligence-based prediction models. Eur Heart J 2023;44:4831–4834. 37897346 10.1093/eurheartj/ehad727 PMC10702458

[65] Wilkinson MD, Dumontier M, Aalbersberg IJJ, Appleton G, Axton M, Baak A, et al The FAIR Guiding Principles for scientific data management and stewardship. Sci Data 2016;3:160018. 26978244 10.1038/sdata.2016.18 PMC4792175

