# Supplementary material for: Prediction of cardiovascular markers and diseases using retinal fundus images and deep learning: a systematic scoping review
Source: Eur Heart J Digit Health. 2024 Sep 10;5(6):660–9. doi: 10.1093/ehjdh/ztae068 (PMC11570365; doi:10.1093/ehjdh/ztae068)
Supplement: ztae068_Supplementary_Data [file ztae068_supplementary_data.pdf]

Electronic Supplementary Materials

Table 1 - Study design and outcome of included studies

	**Markers of subclinical CVD**	**Clinical CVD**
**Cross-sectional studies**	systolic and diastolic blood pressure[34,38,41,42]<br>left ventricular characteristics[32]<br>brachial-ankle pulse-wave velocity[40]<br>coronary artery calcium score[27,29,43,44,46,47]<br>carotid intima-media thickness[28]	hypertension[31,45]<br>peripheral arterial disease[39]<br>coronary heart disease[17,33]<br>cerebrovascular disease[17]<br>stroke[30,35–37]<br>diagnosis of CVD[26]
**Follow-up studies**		major adverse cardiovascular events[41]<br>peripheral arterial disease[38]<br>coronary heart disease[38,47]<br>stroke[38,46–48]<br>myocardial infarction[32,38,46,48]<br>major heart failure[46,48]<br>CVD mortality[28,43,46,47]
